# Supplementary material for: Highly wear-resistant and low-friction Si3N4 composites by addition of graphene nanoplatelets approaching the 2D limit
Source: Sci Rep. 2017 Aug 30;7:10087. doi: 10.1038/s41598-017-10290-5 (PMC5577236; doi:10.1038/s41598-017-10290-5)
Supplement: Supplementary file 1 — Supplementary Information [file 41598_2017_10290_MOESM1_ESM.pdf]

Supplementary Information

Highly wear-resistant and low-friction Si_3N_4 composites by addition of graphene nanoplatelets approaching the 2D limit

*Orsolya Tapasztó, Jan Balko, Viktor Puchy, Péter Kun, Gergely Dobrik, Zsolt Fogarassy, Zsolt Endre Horváth, Jan Dusza, Katalin Balázsi, Csaba Balázsi, Levente Tapasztó**

1. Raman analysis of the FL-GNP thickness

We have performed additional analysis of the Raman spectra of FL-GNP flakes prepared by ball milling with melamine addition. A typical Raman spectrum acquired on the FL-GNP exfoliated by melamine addition is shown in Fig. S1. Several methods have been suggested in the literature to estimate the layer number based on the Raman spectrum. The shape of the 2D peak is one of the most widely used methods, as discussed in the main text. Also the shape of the D peak can be indicative for the layer number. For bulk graphite (>10 layers) the D peak has two Lorentzian components, while for few-layers it can be fitted by a single Lorentzian. This is clearly the case for our FL-GNPs prepared by melamine addition (Fig. S1). Another measure often used for the estimation of layer number is the full width at half maximum (FWHM) of the 2D peak, which is typically in the range of 60-70 cm^{-1} in our FL-GNP samples. The layer number estimated based on this is again below 10 layers[1,2].

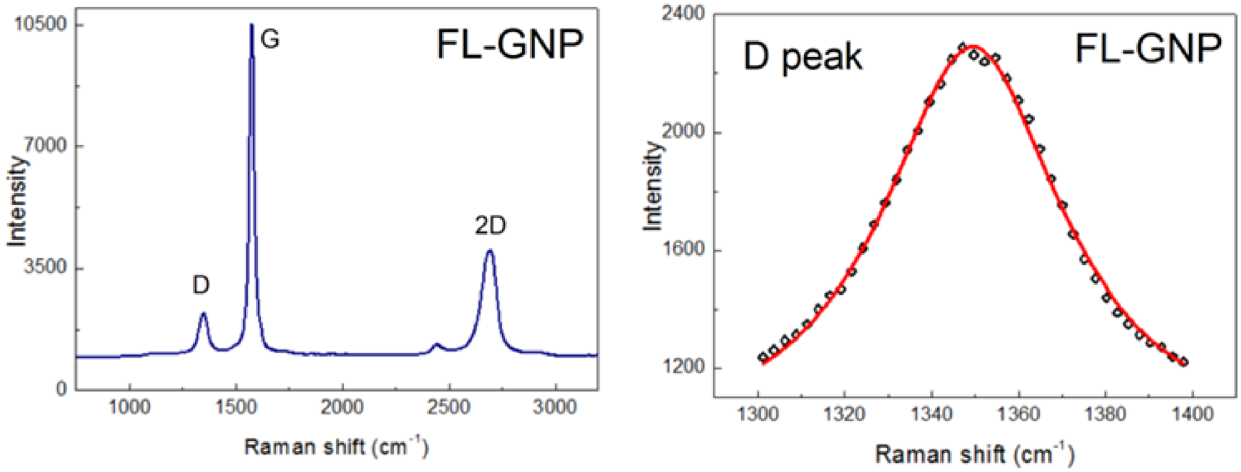

Fig. S1. *Characteristic Raman spectrum of FL-GNPs exfoliated by milling in the presence of melamine displaying a D peak that can be fitted by a single Lorentzian.*

The intensity ratio of the G and 2D peaks is also used for estimating the layer thickness. However, in our case this parameter did not provide a reproducible estimate, as spectra with largely varying I 2D / I G intensity ratios have been measured (Fig. S2), depending on the location. However, it is well-known that this ratio can be strongly influenced by other factors than layer number, such as doping or strain.

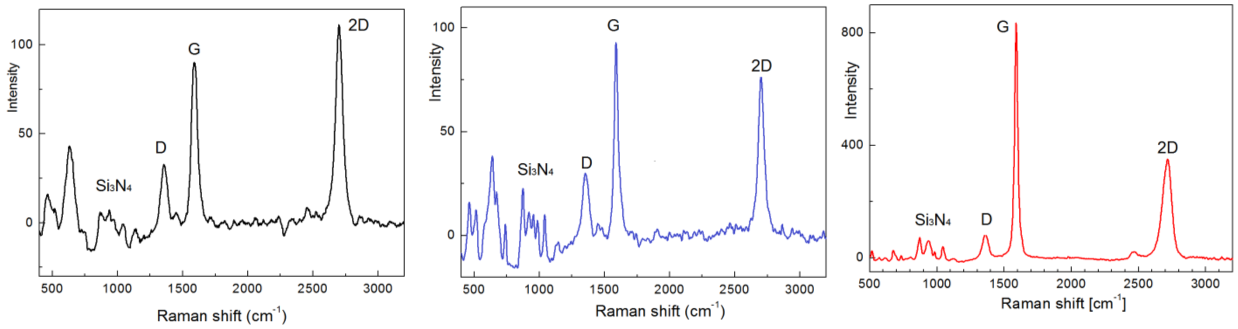

Fig.S2. *Raman spectra acquired at the wear tracks displaying various I2D/IG ratios*

From the Raman investigations we conclude that some areas of the wear tracks are covered by individual FL-GNPs with a thickness below 10 layers, while other areas are covered by similar FL-GNPs, but overlapping of each other. This can lead to a decrease I 2D / I G ratio; however, the shape of the 2D (and D) peaks still clearly indicates the few-layer nature of the GNPs.

Raman measurements can also identify possible melamine residues. In Figure S3 below, we compare Raman spectra acquired on a FL-GNP sample (before sintering) with melamine residue and another from which melamine was removed during washing with hot water.

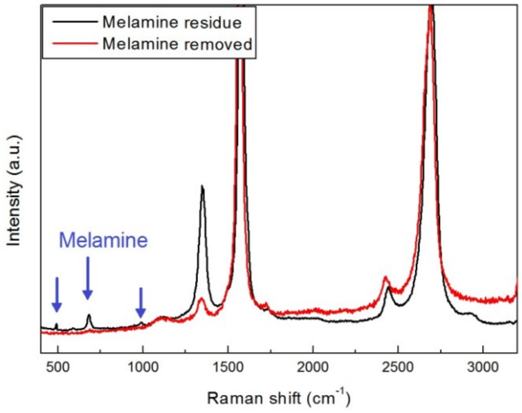

Fig. S3. *Raman spectra of FL-GNPs with and without melamine residues after washing with hot water.*

The samples containing melamine display a larger D peak, due to additional scattering, originating from the melamine. However, most importantly, melamine is completely removed from the final composite materials, as it decomposes above 350 C and the samples were sintered

at 1600 C. In accordance with this, the Raman (and XRD) spectra taken on the sintered composites never show the signature of melamine.

2. XRD Measurements

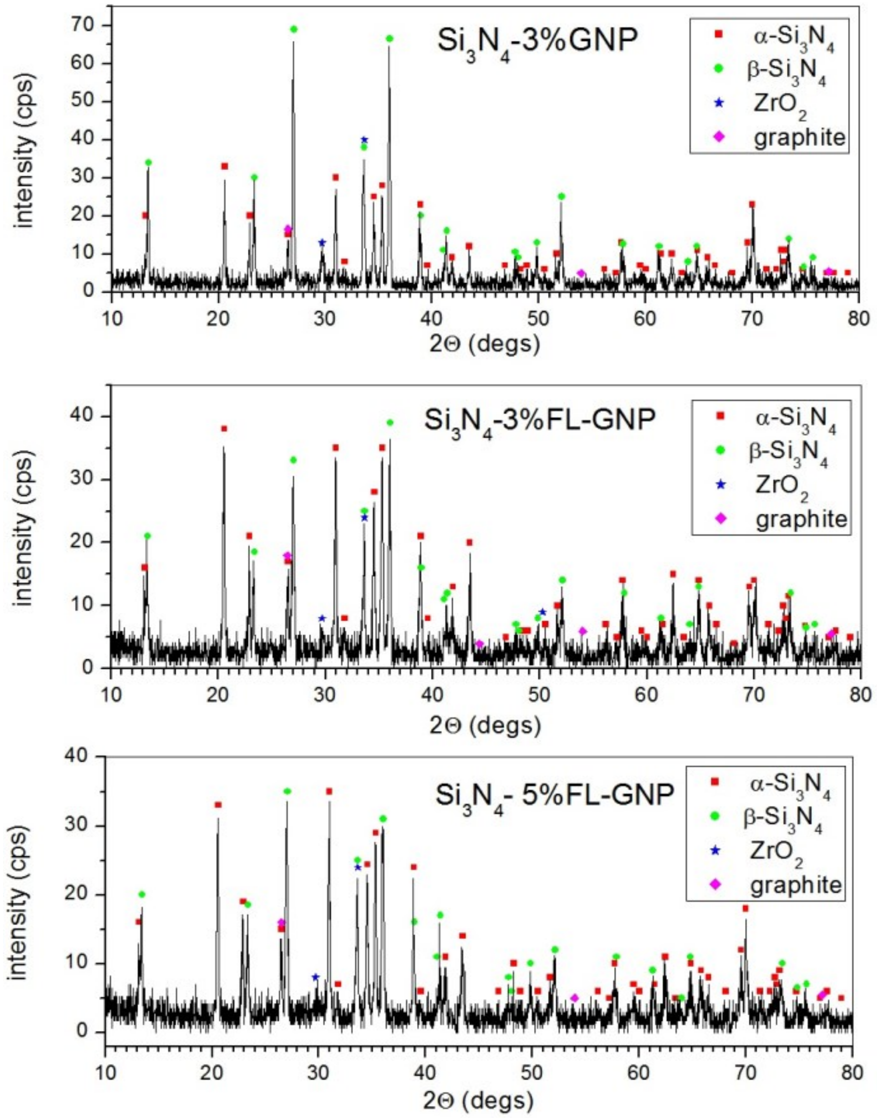

Fig. S4. *Characteristic XRD spectra of the composites*

XRD measurements have been employed to reveal the phase composition of the composites. Besides alpha and beta Si_3N_4 phases, and graphitic carbon (FL-GNP), XRD data also indicate the presence of small amounts of ZrO_2 in the range of 1.1 – 2.4 w t%, which can be attributed to residues form the milling with ZrO_2.balls

3. Optical microscopy of wear tracks

Optical microscopy investigations also revealed clear differences between the wear tracks of the 3% GNP and 3% FL-GNP samples.

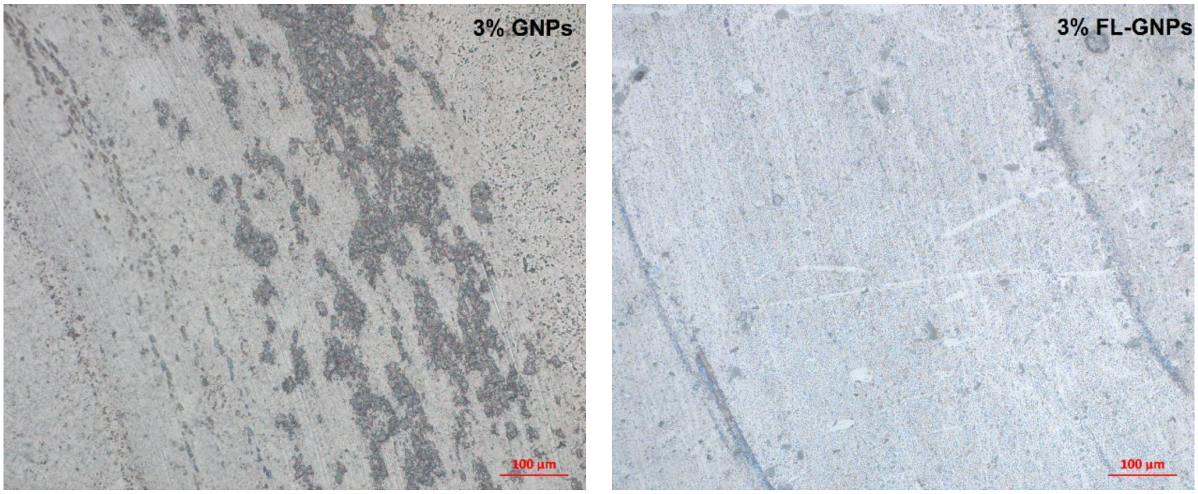

Fig.S5. *Optical images of the wear tracks*

While the GNP sample is characterized by large abrasion marks of order of 100 microns, in the case of 3% FL-GNP samples wear marks of less than 10 microns were typically observed. We attribute this to the lack of a continuous tribo-film formation in both cases; however, for 3% FL-GNP sample the coverage - although not continuous - it is still much more homogeneous than for the 3% GNP sample, due to the much larger surface area of the thinner flakes.

4. SEM imaging of counterpart ball wear

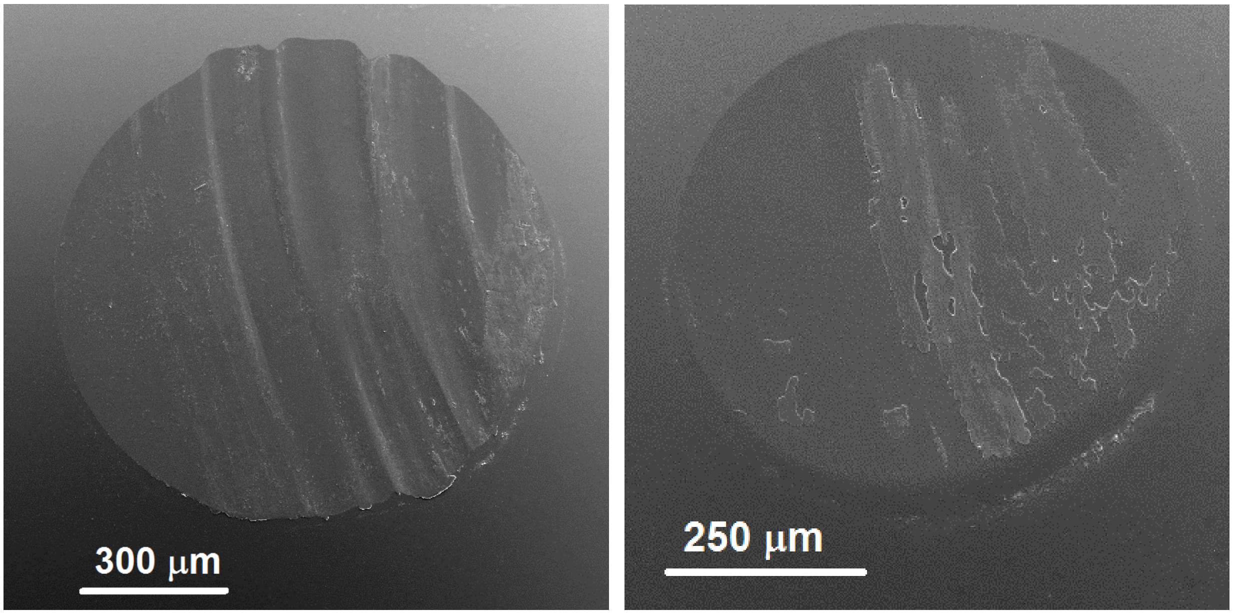

Fig.S6. *Low magnification SEM images of the wear marks on counterpart Si_3N_4 balls probingr the monolithyc Si_3N_4 sample (left) and the 5 wt% FL-GNP/Si_3N_4 sample (right).*

The counterpart Si_3N_4 ball probing the reference Si_3N_4 sample, displays pronouncedr wear tracks, which we attribute to abrasive wearing as also evidnced on the sample surface (Fig.4). By contrast the surface of the Si_3N_4 ball sliding on the 5 wt%FL-GNP/ Si_3N_4 sample remains relatively smooth. In the latter case, the marks on the SEM image can be attributed to the adhesion of the FL-GNP based tribo-film protecting also the ball surface. This finding is further confirmed by energy-dispersive x-ray spectroscopy (EDS) measurements, revealing a clear carbon peak on the surface of this ball.

5. SEM imaging of wear tracks

Imaging graphene nanoplatelets with SEM after the wearing process inside the wear track is a challenging task. Thicker nanoplatelets or larger FL-GNP agglomerates are easier to identify in SEM images, while smaller a thinner flakes remain hard to observe.

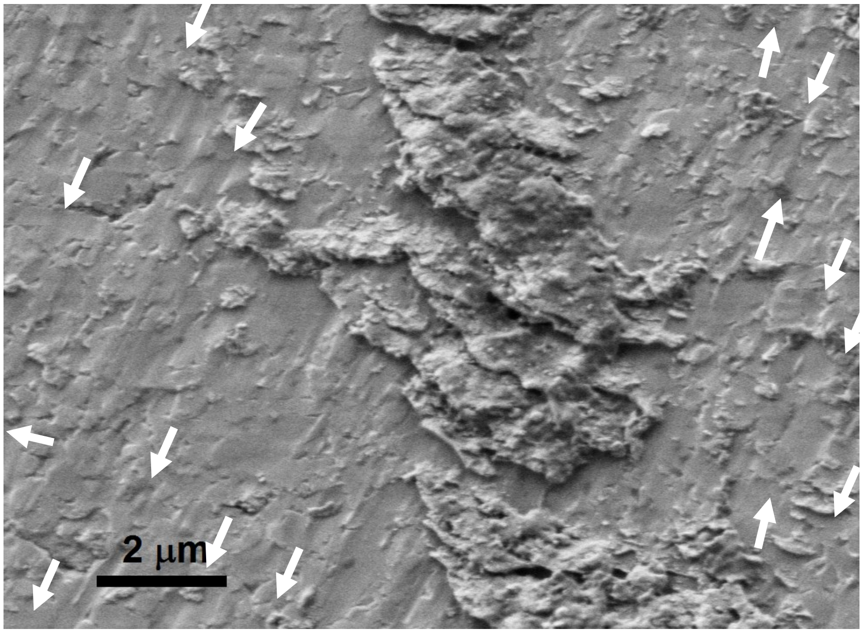

Fig.S7. *Scanning Electron Microscopy image of the wear track for 5wt% FL-GNP/Si_3N_4 sample displaying large FL-GNP aggregates (middle) along with smaller flakes indicated by arrows*

In the higher resolution SEM image acquired within the wear track of the 5% FL-GNP sample displayed above, one can easily identify large graphene nanoplatelet agglomerates in the central part of the image. However, upon more careful examination, thinner and smaller lateral size flakes can also be identified (some of them marked by arrows). Energy dispersive x-ray spectroscopy (EDS) measurements confirmed the elevated carbon content on features identified as graphene nanoplatelets in the wear track. The SEM images of FL-GNPs within the wear tracks are in good agreement with the Raman mapping results (Fig.6), indicating the varying thickness of the FL-GNP based tribo-film, with thicker aggregates (clearly identifiable in SEM images) appearing bright red in the Raman maps, while thinner FL-GNP s, barely visible in the SEM, correspond to the darker red areas of the Raman maps.

Supplementary references

[1] Hao Y et al. Probing layer number and stacking order of few-layer graphene by Raman spectroscopy Small 6, 195 (2010),
[2] Leon V et al. Production and stability of mechanochemically exfoliated graphene in water and culture media Nanoscale 8, 14548 (2016)
